# Density of medical and recreational cannabis outlets: racial/ethnic differences in the associations with young adult intentions to use cannabis, e-cigarettes, and cannabis mixed with tobacco/nicotine

**DOI:** 10.1186/s42238-021-00084-y

**Published:** 2021-07-09

**Authors:** Regina A. Shih, Joan S. Tucker, Eric R. Pedersen, Rachana Seelam, Michael S. Dunbar, Aaron Kofner, Caislin Firth, Elizabeth J. D’Amico

**Affiliations:** 1 RAND Corporation, 1776 Main St., Santa Monica, CA 90407 USA; 2 Department of Psychiatry and Behavioral Sciences, Keck School of Medicine, University of Southern California, Los Angeles, USA; 3 Faculty of Health Sciences, Simon Fraser University, Burnaby, B.C. Canada

**Keywords:** Recreational cannabis, Medical cannabis, Marijuana, E-cigarettes, Cannabis/tobacco co-use, Cannabis dispensary, Cannabis retailers, Density, Young adults

## Abstract

**Background:**

Differences in access to medical versus recreational cannabis outlets and their associations with intentions to use cannabis have not yet been examined among young adults. This study compares the associations between densities of medical versus recreational cannabis outlets and young adults’ intentions to use cannabis, electronic cigarettes, and cannabis mixed with tobacco/nicotine products. Racial/ethnic differences in these associations were examined.

**Methods:**

Young adults ages 18–23 (mean age = 20.9) in Los Angeles County were surveyed online in 2018 after the legalization of recreational cannabis (*n* = 604). Multiple linear regressions were estimated for the entire sample and stratified by race/ethnicity. Outcomes were intentions to use cannabis, electronic cigarettes, and cannabis mixed with tobacco/nicotine in the next 6 months. Density was measured as the number of medical cannabis dispensaries (MCDs), recreational cannabis retailers (RCRs), and outlets of any type within 5 miles of respondents’ homes.

**Results:**

Living near more outlets of any type was not significantly associated with intentions to use in the full sample, adjusting for individual- and neighborhood-level characteristics. However, race/ethnicity-stratified models indicated that living near more outlets of any type and more RCRs were significantly associated with stronger co-use intentions among white young adults. Higher MCD density was marginally associated with stronger co-use intentions among Asian young adults. However, higher MCD density was significantly associated with lower intentions to use e-cigarettes among Hispanic young adults.

**Conclusions:**

The results suggest racial/ethnic differences in the impact of living near cannabis outlets on intentions to use. Prevention efforts targeting young adults who live near more cannabis outlets may be especially beneficial for white and Asian young adults.

## Background

### Recreational cannabis legalization and young adult intentions to use cannabis

Recreational cannabis is now available for legal purchase among adults 21 years and older in 15 states and three territories (National Conference of State Legislatures 2021). Legalization could increase young adults’ use of cannabis through reductions in the price of cannabis, reinforcing normative beliefs about the acceptability of recreational use (Koval et al. 2019), reductions in perceived risks (Anderson and Rees 2014; Fleming et al. 2016), and increased availability and advertising of recreational cannabis. It is important to understand the implications of recreational cannabis access on intentions to use cannabis, which is a key predictor of future use (Andrews and Peterson 2006). This issue is particularly relevant to young adults. A recent review (Carliner et al. 2017) reported that young adults ages 18–29 have the highest prevalence rate of past-year cannabis use (Hasin et al. 2015; Pacek et al. 2015), have greater increases in prevalence over time, and have the sharpest increases in cannabis use (Azofeifa et al. 2016; Compton et al. 2016; Pacek et al. 2015), compared to older age groups (Mauro et al. 2017). At the same time, people ages 18 and older are exhibiting decreases over time in the perceived harmfulness of cannabis (Azofeifa et al. 2016; Compton et al. 2016; Pacek et al. 2015). Therefore, the first aim of this study is to examine how the density of recreational cannabis retailers (RCRs) near where young adults live is associated with their future intentions to use cannabis.

### Access to recreational cannabis versus access to medical cannabis

Sale and possession of recreational cannabis were only recently legalized (first in 2012); thus, few studies have compared the effects of increasing the density of RCRs on medical cannabis dispensaries (MCDs). Increasing density of MCDs has been cross-sectionally linked with greater cannabis use and stronger positive beliefs about cannabis among adults ages 18 and older (Freisthler and Gruenewald 2014) and young adults ages 18–22 (Shih et al. 2019). The density of RCRs is likely to have different effects on individuals’ attitudes towards cannabis because recreational cannabis legalization has wider impacts on the pricing and availability of cannabis (Hunt and Pacula 2017), whereas medical cannabis is only available for legal purchase from MCDs among a subset of the population enrolled in a state’s medical cannabis program (National Conference of State Legislatures 2021). California was the first state to legalize and establish the medical cannabis program in 1996 and 2005, respectively. Proposition 64 (Adult Use of Marijuana Act) legalized recreational cannabis for adults (21 + years old) through voter initiative in November 2016. On January 1, 2018, state licensed retail outlets began operating in California to sell non-medical cannabis products. The second aim of this study therefore compares how intentions to use cannabis among young adults in California are associated with the density of recreational versus medical outlets near where they live.

### Co-use of cannabis with tobacco/nicotine

Co-use of cannabis and tobacco/nicotine among adults in California, and the USA more generally, has increased in recent years (D’Amico et al. 2016a, b; Schauer et al. 2015). Cannabis and tobacco/nicotine can be co-used either by co-administration of both products through the same delivery device or by using both products sequentially one right after another so that their effects overlap. It is likely that increases in rates of electronic cigarette (e-cigarette) use, availability of electronic nicotine delivery systems (ENDS) (Center for Public Health Systems Science 2016; Park et al. 2020), and availability of recreational cannabis through recreational legalization have jointly contributed to the increases in co-use of cannabis with tobacco/nicotine. The third aim of our study therefore tests the hypothesis that RCR density is associated specifically with the intention to co-use cannabis and tobacco/nicotine (e.g., mixing cannabis oil and nicotine e-juice in ENDS), in addition to intentions to use each substance separately.

### Racial/ethnic differences in associations between cannabis outlet density and young adult cannabis use

Some epidemiological studies suggest that the risk of cannabis use differs by racial/ethnic group. For example, the prevalence of past-year cannabis use has increased more for non-Hispanic white individuals than other racial/ethnic groups (Shmulewitz et al. 2017). In terms of attitudes towards cannabis, non-Hispanic white adolescents are more likely to report intentions to use cannabis relative to non-Hispanic black and Hispanic adolescents (Palamar et al. 2014). Similarly, non-Hispanic white individuals show stronger declines in perceptions of the risks of cannabis use over time compared to non-Hispanic black or Hispanic individuals (Pacek et al. 2015). Yet, the one study that examined legalization effects on intentions to use did not find differences between non-Hispanic white and Hispanic adolescents (Rusby et al. 2018), perhaps because of their small sample size (*n* = 444) and focus on middle school adolescents, which does not include those who are of legal purchasing age. Young adults are important to study because they may navigate their neighborhoods in different ways than do adolescents; for example, young adults may drive further for work and school, so measures of access should account for larger geographic regions for young adults who are relatively understudied in comparison with adolescents.

We posit that increased density of cannabis outlets may differentially influence intentions to use by race/ethnicity among young adults. Persons of color and those with lower socioeconomic status may also have greater access to cannabis because MCDs (Morrison et al. 2014; Thomas and Freisthler 2016; Tabb et al. 2018) and RCRs (Shi et al. 2016; Firth et al. 2020) are geographically more concentrated in neighborhoods with higher racial/ethnic minority populations and higher economic poverty. Thus, our fourth and final aim examines the racial/ethnic differences in how the densities of RCRs and MCDs near where young adults live are associated with their intentions to use cannabis, e-cigarettes, and cannabis mixed with tobacco/nicotine.

## Methods

### Aim of the present study

This study focused on a sample of young people ages 18 to 23 in Los Angeles (L.A.) County, surveyed in 2018, to compare the associations between the density of RCRs and MCDs in one’s neighborhood and individual-level intentions to use e-cigarettes, cannabis, or cannabis mixed with tobacco/nicotine products, and whether differences exist by race/ethnicity.

### Design, setting, and participants

Participants originated from two cohorts of students in sixth and seventh grade in 2008, who were recruited from 16 middle schools from three school districts in Southern California (D’Amico et al. 2012). Schools were initially selected to obtain a racially/ethnically diverse sample and to have similar substance use rates at baseline. Participants completed annual surveys at wave 1 (fall 2008) through wave 5 (spring 2011) which were administered during physical education classes. Adolescents transitioned from the 16 middle schools to over 200 high schools following wave 5 and were subsequently re-contacted and re-consented to complete annual web-based surveys, for which they were compensated. This analysis utilizes data from a single wave (wave 10) when participants were age 20.7 years on average. At wave 10, we retained 90% of the sample from wave 9, similar to the retention rates at earlier waves (D’Amico et al. 2018). Demographics and substance use at wave 9 did not predict attrition from wave 9 to wave 10, similar to what we have found at earlier waves (D’Amico et al. 2016a, b).

Wave 10 was chosen because its data collection window (July 2017 through June 2018) coincides with the dates during which we collected data on the availability of RCRs, which could legally sell recreational cannabis (if licensed) as of January 2018. Of the 869 participants who completed wave 10 after January 2018, we geocoded residential addresses to census tract, longitude, and latitude using ArcGIS (ArcMap, version 10.4. 2018) for 708 (81%) participants without P.O. boxes or missing/incomplete addresses. Of these, we excluded 102 (14%) who did not live in L.A. County and two (0.3%) who were out of the age range (i.e., < 18), resulting in a final analytic sample of 604. Almost 73% of this analytic sample reported being a college student, of whom 10% reported living on-campus; thus, dorm addresses were included for the purpose of geocoding for this subset of respondents.

### Measures

#### Future intentions to use cannabis, use e-cigarettes, and mix cannabis and tobacco/nicotine

Because the frequency of past month co-use is relatively low in this sample, we focus on intentions to use cannabis, intentions to use e-cigarettes, and intentions to co-use cannabis with tobacco/nicotine. Participants were asked separate questions about whether they would use marijuana, use an e-cigarette, or mix tobacco/nicotine and marijuana in the next 6 months (4 = *definitely yes*, 3 = *probably yes*, 2 = *probably no*, 1 = *definitely no*), which we heretofore refer to as intentions to use cannabis, intentions to use e-cigarettes, and intentions to mix cannabis with tobacco/nicotine, respectively. For the latter item, “mixing tobacco and marijuana” was defined as using both at the same time through the same delivery device (e.g., the same joint, bowl pack, vaporizer cartridge).

#### Density of MCDs and RCRs

We searched for and collected locations of MCDs and RCRs that were open in L.A. County with a physical address (excluding delivery only MCDs) from two websites: WeedMaps and Leafly. We then verified the address data and confirmed that each outlet was open for business through a series of procedures, such as calling the outlets and reviewing recent Google Maps imagery of the storefront. We confirmed whether each outlet was licensed or unlicensed to sell cannabis through a comparison with datasets from the City of L.A. Department of Cannabis Regulation and the California Bureau of Cannabis Control. This represents a snapshot of MCDs and RCRs within neighborhoods at a specific time point (March/April 2018) and was the best-known feasible method for collecting licensed and unlicensed MCD and RCR information for research purposes (Pedersen et al. 2018).

Outlets were geocoded to their latitude and longitude. To derive a measure of density, we obtained L.A. County-specific travel distances. This was calculated using the Network Analyst extension of ArcMap version 10.4. A 10-min drive service area was constructed around each residential address. Then, the network distance between the location and a random sample of nodes of the service area were measured and averaged to determine that 5 miles was the average distance that residents in L.A. County could travel in 10 min. This size buffer was motivated by studies showing that the average driving distance in the USA for consumer goods is approximately 10 min (Hamrick and Hopkins 2012; Ver Ploeg et al. 2015). We drew a 5-mile circular buffer around respondents’ home addresses and counted the number of (1) total outlets, (2) dispensaries that sold only medical cannabis (MCDs), and (3) retailers that sold any recreational cannabis (RCRs), including if they sold both medical and recreational cannabis.

Prior work in this area indicates that positive beliefs about cannabis are positively correlated with outlets’ storefront signage indicating that cannabis is being sold (Shih et al. 2019). Thus, we only included outlets with obvious signage as determined by (a) the name of the store clearly indicating it sold cannabis (e.g., The Pot Joint), (b) a marijuana leaf or paraphernalia picture being displayed, or (c) the word “marijuana” or a clear variant of it appearing in the storefront signage (above and beyond similar wording in the store’s name). We used Google Maps Street View and a variety of websites and social media to review consumer- and store owner-posted pictures (and timestamps) to observe signage indicating that the store sold cannabis (Pedersen et al. 2018).

#### Neighborhood-level education

We controlled for census tract-level educational attainment greater than bachelor’s degree among those ages 25 and older, using the 2014–2018 5-year averages from the American Community Survey.

### Statistical analysis

We conducted multiple linear regressions in SAS (SAS/ACCESS® 9.4. 2013) to estimate the association between the count of all outlets, MCDs, and RCRs within a 5-mile radius and each outcome in separate models. Models adjusted for age, gender, race/ethnicity, educational attainment, employment (with non-Hispanic white, male, under age 21, never attended/not currently in college, and unemployed as the reference groups), and neighborhood-level educational attainment with census tract-level cluster robust standard errors. We ran these models for the entire sample and then stratified by race/ethnicity. We also tested interaction terms between each measure of RCR and MCD density and race/ethnicity.

## Results

### Sample descriptive characteristics

Approximately 49% (293) of the sample was female, 74% (445) were currently in high school or college, 54% (329) of the sample was Hispanic, 19% were white (117), and 14% (85) were Asian (Table 1). The average number of MCDs and RCRs located within 5 miles of respondents’ home addresses was 3.1 (SD = 1.5) and 9.1 (SD = 8.4), respectively.

**Table 1:** Sociodemographic and cannabis-related characteristics of n = 604 online survey respondents in Los Angeles County

**Covariates**	
Age in years, *mean (SD)*	20.87 (0.74)
Female gender, *% (n)*	293 (48.51%)
Educational attainment, *% (n)*	
Currently in high school or college	445 (73.68%)
Graduated from college or technical/trade school	28 (4.64%)
Other	131 (21.69%)
Race/ethnicity, *% (n)*	
Hispanic	329 (54.47%)
Non-Hispanic white	117 (19.37%)
Non-Hispanic Asian	85 (14.07%)
Non-Hispanic Other/multi-racial	73 (12.09%)
Employment status, *% (n)*	
Employed	416 (68.99%)
Unemployed	187 (31.01%)
Census tract-level percent of educational attainment greater than a bachelor’s degree among adults 25 and older, *mean (SD)*	40.37% (19.08%)
**Independent variables**	
Number of total cannabis outlets within 5 mi of individual’s home address, *mean (SD), median, inter-quartile range*	12.61 (10.07), 10.00, 10.00
Number of medical cannabis dispensaries within 5 mi of individual’s home address, *mean (SD), median, inter-quartile range*	3.13 (1.45), 3.00, 2.00
Number of recreational cannabis retailers within 5 mi of individual’s home address, *mean (SD), median, inter-quartile range*	9.06 (8.37), 7.00, 8.00
**Dependent variables**	
Future intentions to use cannabis (range from 1 to 4), *mean (SD)*	2.21 (1.19)
Future intentions to use electronic cigarettes (range from 1 to 4), *mean (SD)*	1.56 (0.90)
Future intentions to mix tobacco/nicotine and cannabis (range from 1 to 4), mean (SD)	1.41 (0.79)

*Note*: Recreational cannabis retailers include stores that sell both medical and recreational cannabis; medical cannabis dispensaries include only stores that sell only medical cannabis

### Associations between MCDs/RCRs and intentions

Among the total sample, the intention to use cannabis, to use e-cigarettes, or to mix cannabis with tobacco/nicotine over the next 6 months was not significantly associated with living near more outlets (Table 2).

**Table 2:** Medical and recreational cannabis outlets within 5 mi and individual intentions to use (full sample, *n* = 604)

	**Coefficient (95% confidence interval), ** ***p*** **-value**		
	**Cannabis**	**Electronic cigarettes**	**Cannabis mixed with tobacco/nicotine**
Count of all dispensaries	0.005 (− 0.005, 0.015), *p* = 0.343	< .001 (− 0.006, 0.007), *p* = 0.857	0.003 (− 0.002, 0.008), *p* = 0.179
Count of medical cannabis dispensaries	0.021 (− 0.044, 0.086), *p* = 0.521	− 0.036 (− 0.078, 0.007), *p* = 0.101	0.012 (− 0.021, 0.045), *p* = 0.480
Count of recreational cannabis retailers	0.006 (− 0.007, 0.018), *p* = 0.362	0.001 (− 0.007, 0.009), *p* = 0.723	0.004 (− 0.002, 0.01), *p* = 0.214

*Notes*: ^†^0.05 < *p* < 0.10; *p ≤ 0.05; ** ≤ 0.01. Coefficients are from a multiple linear regression and represent associations between outlet density and individual-level intentions to use among adults 18 and older living in Los Angeles County in 2018. Models were cluster-robust and adjusted for age, gender, race/ethnicity, educational attainment, employment, and neighborhood educational attainment

### Racial/ethnic differences in associations

When models were stratified by race/ethnicity, several findings emerged. Table 3 indicates that among non-Hispanic white young adults, the density of any type of outlet was positively associated with stronger future intentions to mix cannabis with tobacco/nicotine (β = 0.018, 95% C.I = 0.007, 0.029). This coefficient can be interpreted as each additional cannabis outlet in a 5-mile range is associated with increasing intentions to mix cannabis with tobacco/nicotine by 0.018 points. The magnitude of effect and significance level was similar to the association of RCRs with intentions to mix cannabis with tobacco/nicotine (β = 0.022, 95% C.I. = 0.007, 0.037). In addition, living near a greater number of MCDs was marginally associated with stronger intentions to co-use cannabis with tobacco/nicotine among Asian young adults (β = 0.187, 95% C.I =  − 0.006, 0.379). However, living near a greater number of MCDs was associated with weaker intentions to use e-cigarettes among Hispanic young adults (β =  − 0.045, 95% C.I. =  − 0.088, − 0.002). Interaction terms (not presented in the tables) showed that Asian young adults had a significantly larger magnitude of association between higher MCD density and stronger intentions to use e-cigarettes compared to white young adults, and Hispanic young adults had smaller magnitudes of association betwen higher density of all outlets and RCRs and lower intentions to co-use cannabis with tobacco/nicotine compared to white young adults.

**Table 3:** Medical and recreational cannabis outlets within 5 mi and individual intentions to use, by young adults’ race/ethnicity

	**Coefficient (95% confidence interval), ** ***p*** **-value**		
	**Cannabis**	**Electronic cigarettes**	**Cannabis mixed with tobacco/nicotine**
**Non-Hispanic white (** ***n*** ** = 117)**			
Count of all outlets	0.015 (− 0.005, 0.035), *p* = 0.139	0.008 (− 0.009, 0.024), *p* = 0.361	0.018 (0.007, 0.029), *p* = 0.002**
Count of medical cannabis dispensaries	0.148 (− 0.024, 0.321), *p* = 0.091^†^	− 0.061 (− 0.205, 0.083), *p* = 0.400	0.07 (− 0.048, 0.188), *p* = 0.241
Count of recreational cannabis retailers	0.015 (− 0.01, 0.04), *p* = 0.228	0.011 (− 0.009, 0.032), *p* = 0.271	0.022 (0.007, 0.037), *p* = 0.005**
**Hispanic (*n*** ** = 329)**			
Count of all outlets	0.001 (− 0.011, 0.013), *p* = 0.896	− 0.003 (− 0.01, 0.003), *p* = 0.323	− 0.001 (− 0.006, 0.004), *p* = 0.767
Count of medical cannabis dispensaries	− 0.010 (− 0.083, 0.064), *p* = 0.796	− 0.045 (− 0.088, − 0.002), *p* = 0.040*	− 0.009 (− 0.042, 0.023), *p* = 0.569
Count of recreational cannabis retailers	0.002 (− 0.014, 0.017), *p* = 0.842	− 0.003 (− 0.011, 0.005), *p* = 0.431	< − 0.001 (− 0.007, 0.006), *p* = 0.836
**Asian (*n*** ** = 85)**			
Count of all outlets	0.02 (− 0.009, 0.048), *p* = 0.168	0.02 (− 0.011, 0.051), *p* = 0.198	0.009 (− 0.02, 0.039), *p* = 0.532
Count of medical cannabis dispensaries	0.203 (− 0.052, 0.457), *p* = 0.116	0.146 (− 0.11, 0.401), *p* = 0.258	0.187 (− 0.006, 0.379), *p* = 0.058^†^
Count of recreational cannabis retailers	0.019 (− 0.017, 0.055), *p* = 0.300	0.02 (− 0.019, 0.059), *p* = 0.303	0.006 (− 0.031, 0.043), *p* = 0.746
**Other race/multi-racial (*n*** ** = 73)**			
Count of all outlets	0.011 (− 0.049, 0.07), *p* = 0.720	0.002 (− 0.041, 0.045), *p* = 0.925	< .001 (− 0.038, 0.040), *p* = 0.963
Count of medical cannabis dispensaries	− 0.014 (− 0.303, 0.274), *p* = 0.920	− 0.106 (− 0.328, 0.116), *p* = 0.342	− 0.045 (− 0.204, 0.114), *p* = 0.573
Count of recreational cannabis retailers	0.013 (− 0.053, 0.079), *p* = 0.698	0.007 (− 0.039, 0.053), *p* = 0.761	0.003 (− 0.041, 0.047), *p* = 0.890

*Notes*: ^†^0.05 < *p* < 0.10; *p ≤ 0.05; ** ≤ 0.01. Coefficients are from a multiple linear regression and represent associations between outlet density and individual-level intentions to use among adults 18 and older living in Los Angeles County in 2018. Models were cluster-robust and adjusted for age, gender, educational attainment, employment, and neighborhood educational attainment

## Conclusions

This is the first study to simultaneously examine the density of both MCDs and RCRs around young adults’ homes and associations with future intentions to use cannabis, including the co-use of cannabis with tobacco/nicotine. Our results suggest that young adults who lived in an area with a greater density of any type of outlet were not significantly more likely to report stronger intentions to use cannabis, e-cigarettes, or cannabis mixed with tobacco/nicotine in the future. One prior study found that associations between legalization and more positive attitudes towards cannabis were stronger among those who had previously used tobacco (Cohn et al. 2016). Similarly, data from a Monitoring the Future study found higher odds of intending to use cannabis were associated with recreational legalization among adolescents already at high risk for use, including cigarette smokers (Palamar et al. 2014). Thus, future research in a larger sample could examine whether the increasing density of RCRs and MCDs may be associated with greater intentions to use cannabis among those who previously used tobacco/nicotine or cannabis.

We found several interesting differences by race/ethnicity that may have important implications for understanding disparities in cannabis use. For example, only white young adults who lived near a higher density of any outlets tended to report stronger intentions to co-use cannabis with tobacco/nicotine. Associations between the density of outlets and co-use intentions among white young adults appear to be driven mostly by access to recreational cannabis, suggesting that the effects of RCRs may be stronger than MCDs and that the emergence of RCRs in L.A. County, which made cannabis legally available to everyone over the age of 21 regardless of medical conditions, appears to be especially influential for white young adults. Among Asian young adults, increasing MCD density appears to be somewhat influential for intentions to use cannabis mixed with tobacco/nicotine. It is unclear why MCDs are influential for Asian young adults whereas any type of outlet, but especially RCRs, are influential for co-use intentions among white young adults. One prior study reported no difference in medical marijuana card ownership by race (Tucker et al. 2019a). Thus, white and Asian young adults may have a greater awareness of RCRs and MCDs, respectively, in their neighborhoods. Since both racial groups experienced significant or marginal associations with intentions to co-use, it is worth noting that co-use is associated with more frequent and problematic use, poorer mental and physical health, and delinquent behaviors (Akbar et al. 2019; Cobb et al. 2018; Hernandez-Serrano et al. 2018; Tucker et al. 2019b) compared to single-product use. Moreover, co-use may incur greater health risks compared to the use of single products such as respiratory problems (Layden et al. 2019; Centers for Disease Control and Prevention 2019), although the long-term health effects of vaping co-use are unknown.

Results also indicated that Hispanic young adults who lived near more MCDs reported weaker intentions to use e-cigarettes, but not necessarily cannabis or cannabis mixed with tobacco/nicotine. The inverse association of MCD density with intentions to use e-cigarettes was not expected, and it is unclear why this might have emerged among Hispanic young adults only. Further research is needed to determine whether this is a robust finding and, if so, to understand the impact of MCD exposure on e-cigarette use in Hispanic young adults.

Taken together, these findings could shed light on the density of RCRs as a contextual risk factor for higher rates of co-use among white young adults and suggest that implementation of targeted intervention efforts among residents who live near more RCRs may be an efficient way to curb cannabis use and co-use of cannabis mixed with tobacco/nicotine, especially among white young adults. It is important to note, however, that black, Hispanic, and Asian youths report more negative consequences such as problems with physical health and poor academic functioning when using cannabis and nicotine/tobacco at the same levels of non-Hispanic white youth (D’Amico et al. 2016a, b; Dunbar et al. 2018; Tucker et al. 2019c). Therefore, even though outlet density does not appear to increase intentions to use among Hispanic young adults, they may still be at risk for poorer outcomes even when using at the same levels as white youth. This may be particularly true for Asian young adults who showed a higher risk of intentions to use e-cigarettes associated with MCD dispensary compared to white young adults.

Findings should be considered in the context of limitations. First, we could not examine the density of specialty vape shops or tobacco outlets. There are no publicly searchable databases of specialty vape shops, and tobacco outlet data do not indicate whether shops sell e-cigarettes or vaping products. Moreover, tobacco outlet data do not include single-owner establishments because of privacy concerns, which undercounts the availability of tobacco shops and, therefore, the availability of e-cigarettes or vaping products. Second, the results may not generalize to populations outside of L.A. County. Lastly, the relatively small number of Black young adults in the sample necessitated including them in the other/multi-racial group, which precluded an examination of differences between MCD and RCR density for black young adults.

In summary, there is evidence from this study that non-Hispanic white and, to a lesser extent, Asian young adults who live near a higher density of cannabis outlets are more likely to report stronger intentions to co-use cannabis with tobacco/nicotine over the next 6 months. Future research could use real-time data captured by methods such as ecological momentary assessments to more precisely determine how youths navigate their environments to travel to and purchase from recreational retailers, how cannabis and e-cigarette advertising may affect co-use intentions, and whether policies that seek to restrict geographic locations and density of recreational retailers could potentially reduce harmful levels of cannabis use and co-use with tobacco/nicotine. These restrictions could modify the risk for negative consequences especially among racial/ethnic subgroups who may be more likely to be influenced by their local cannabis retail environments.

## Data Availability

The datasets generated and/or analyzed during the current study are not publicly available due to privacy and confidentiality limitations specified in our IRB approval but may available from the corresponding author on reasonable request.
